# Interoceptive Accuracy in Youth with Tic Disorders: Exploring Links with Premonitory Urge, Anxiety and Quality of Life

**DOI:** 10.1007/s10803-018-3608-8

**Published:** 2018-05-18

**Authors:** Victoria Pile, Jennifer Y. F. Lau, Marta Topor, Tammy Hedderly, Sally Robinson

**Affiliations:** 1 King’s College London, Department of Psychology, Institute of Psychiatry Psychology and Neuroscience, De Crespigny Park, London, SE5 8AF UK; 2 Tic and Neurodevelopmental Movements Service (TANDeM), Children’s Neurosciences Centre, Evelina London Children’s Hospital, Guys and St Thomas’ NHS Foundation Trust, St Thomas’ Hospital, Westminster Bridge Road, London, SE1 7EH UK; 3 Paediatric Neuropsychology Service, St Georges University Hospitals NHS Foundation Trust, St Georges Hospital, London, SW17 0QT UK

**Keywords:** Tourette syndrome, Anxiety, Heartbeat perception, Interoceptive awareness, Tic disorders

## Abstract

Aberrant interoceptive accuracy could contribute to the co-occurrence of anxiety and premonitory urge in chronic tic disorders (CTD). If it can be manipulated through intervention, it would offer a transdiagnostic treatment target for tics and anxiety. Interoceptive accuracy was first assessed consistent with previous protocols and then re-assessed following an instruction attempting to experimentally enhance awareness. The CTD group demonstrated lower interoceptive accuracy than controls but, importantly, this group difference was no longer significant following instruction. In the CTD group, better interoceptive accuracy was associated with higher anxiety and lower quality of life, but not with premonitory urge. Aberrant interoceptive accuracy may represent an underlying trait in CTD that can be manipulated, and relates to anxiety and quality of life.

## Introduction

Chronic tic disorders (CTD) are heritable and impairing neuropsychiatric conditions, typically with onsets in early childhood (Ganos et al. 2015). While tics are considered the main feature of CTD, the majority of people with CTD report a premonitory urge (PU), which is an uncomfortable sensation before tic onset, relieved by expressing the tic. PU is associated with quality of life (Crossley and Cavanna 2013) and, compellingly, 71% of adults report they would not tic if the urge was not present (Kwak et al. 2003). Yet, the mechanisms underlying PU are poorly understood, especially in children. Another significant feature of CTD is anxiety (Bitsko et al. 2015). As well as being very common in youth with CTD, anxiety persists even if tics wane and may moderate the relationship between tic severity and functional impairment (Lewin et al. 2011). Despite the close and detrimental relationship between tics and anxiety, research investigating mechanisms linking them is sparse. One factor implicated independently in PU and anxiety is interoception. Khalsa and Lapidus (2016) define interoception as “the process of how the brain senses and integrates signals originating from inside the body, providing a moment by moment mapping of the body’s internal landscape”. One study has investigated interoceptive accuracy (IA) in adults with CTD to explain PU, but no studies have investigated IA in childhood CTD or used IA to consider why youth with CTD are at enhanced risk of anxiety. The current study addressees these limitations by exploring IA and its relationship to PU and symptoms of anxiety in youth with CTD, with the aim of informing clinical treatments.

Interoception is multifaceted and includes information processing that is conscious and non-conscious. Garfinkel et al. (2015) distinguish *interoceptive accuracy* (“performance on objective behavioural tests of heartbeat detection”) from *interoceptive sensibility* (“self-evaluated assessment of subjective interoception”) and *interoceptive awareness* (“metacognitive awareness of IA”). Here, we investigate a general marker of IA to provide insight into the ability to gauge internal bodily signals accurately. This is distinguished from the cognitive interpretation (or indeed catastrophic misinterpretation) of these signals, for example as indicating an oncoming tic or a heart attack. Heartbeat detection tasks are frequently used to quantify individual differences in IA, which is partly pragmatic as heartbeats are frequent and discrete internal events that are easily measured (Garfinkel et al. 2015). The mental tracking paradigm (Schandry 1981) has been extensively used to measure heartbeat perception (HBP) and is considered to produce the most consistently replicable results (Eley et al. 2004). In this paradigm, participants are asked to count their heartbeats for a certain period of time, with the counted number of heartbeats compared to electrocardiogram (ECG) record to gauge accuracy.

The only study of HBP in CTD has been conducted in adults (Ganos et al. 2015). They found that heightened HBP was associated with greater PU, with HBP identified as a better indicator of PU than tic severity or obsessive–compulsive symptoms. The authors proposed that PU represents enhanced (but unhelpful) IA—interestingly, similar brain regions have been implicated in both PU and IA (Craig 2003; Jackson et al. 2011). In contrast to predictions, however, the CTD group had lower HBP than controls. The authors suggested that participants may have down-regulated their interoception to manage PU. Furthermore, Ganos et al. (2015) did not find associations between HBP and other clinical features, such as anxiety. This is in contrast to studies in the general population, where heightened awareness of bodily arousal (alongside misattributions of what this arousal signifies) is proposed to contribute to and maintain anxiety across the age range (Domschke et al. 2010; Dunn et al. 2010; Eley et al. 2007; Stevens et al. 2011).

To date, no studies have explored HBP in youth with CTD, but it is plausible that aberrant HBP could contribute to both difficulties with PU and anxiety. If IA is aberrant in youth with CTD and associated with anxiety, it could offer a novel intervention target to address common co-morbidities in CTD. For IA to be a valid treatment target, it is vital that it can be experimentally manipulated. Woods et al. (2005) suggest that a *lack* of early awareness of PU results in tic expression, which is consistent with the Ganos et al. (2015) finding that the CTD group had lower HBP compared to the control group. Indeed, current psychological interventions for tics aim to enhance awareness (and tolerance) of internal sensations to prevent tic expression (McGuire et al. 2014; Van de Griendt et al. 2013). Whilst it is important to distinguish HBP from PU (as HBP offers a general marker of IA and not a tic-specific marker), HBP provides a well-established paradigm to investigate whether IA is aberrant and can be manipulated in youth with CTD. Experimental attempts to alter IA in the general population have mostly been ineffective (e.g. Khalsa et al. 2008; Stevens et al. 2011), though some recent research has increased HBP using experimental manipulations (Ainley et al. 2012, 2013). Yet, no research has investigated whether IA can be manipulated in youth with CTD.

The current study sought to extend findings from non-tic populations (Domschke et al. 2010; Eley et al. 2007; Stevens et al. 2011) and adults with CTD (Ganos et al. 2015), by exploring IA and its relationship to PU and symptoms of anxiety in youth with CTD. Depression/dysphoria is associated with reduced IA and has been suggested as an explanation for mixed findings (Dunn et al. 2007; Pollatos et al. 2009); inattention is commonly reported for individuals with CTD (Bloch and Leckman 2009) and likely to impact on performance. Therefore, controlling for these symptoms is important. Finally, reduced quality of life has been associated increased anxiety and PU (Conelea et al. 2011; Crossley and Cavanna 2013; Eapen et al. 2016; Hesapçıoğlu et al. 2014). As such, identifying whether HBP relates to quality of life in youth with CTD is of interest, especially if interoceptive awareness is to be considered as a possible transdiagnostic treatment target.

Here we used the experimental task described above (HBP) to investigate IA under two conditions (1) HBP Baseline and (2) HBP Manipulation. The first condition followed previous protocols (Eley et al. 2004; Ganos et al. 2015), whilst in the second condition participants were instructed not to move whilst counting their heatbeats. The aim of this was to artificially enhance self-awareness and mirror situations where youth with CTD are asked to suppress tics. We anticipated that this would result in youth with CTD exhibiting fewer movements in condition 2 than condition 1. To ensure group differences in HBP were not the result of co-morbidities; we controlled for anxiety, depression and inattention, and explored the relationship between these clinical variables and IA. In line with the adult literature, we predicted that (1) HBP at baseline would be lower in youth with CTD relative to matched typically developing controls, and (2) HBP would increase between the HBP Baseline and HBP Manipulation conditions for youth with CTD, but not controls. The HBP Baseline condition was also used to investigate whether enhanced HBP was associated with increased PU, elevated levels of anxiety or poorer tic-related quality of life for youth with CTD.

## Methods

### Participants

Fifty-seven participants completed the study. Three participants were excluded due to equipment failure (n = 1), not understanding instructions (n = 1) and being a control participant with significant tics (n = 1). This resulted in 29 participants with CTD (14 female; age: $\bar{\rm x}$ = 11.27, SD = 1.68) and 25 controls (12 female; age: $\bar{\rm x}$ = 11.17, SD = 2.46). In the CTD group, 24 participants had a diagnosis of Tourette syndrome and five a diagnosis of another CTD. Eight participants in the CTD group had additional diagnoses, including autism spectrum disorder (n = 3); attention deficit hyperactivity disorder (ADHD) (n = 5); obsessive compulsive disorder only (n = 3). These figures include two participants who had diagnoses of both ADHD and ASD and one participant diagnosed with ASD, ADHD and OCD. In terms of medication at the time of completing the study, four participants were prescribed clonidine and two prescribed aripiprazole. IQ and demographic factors were recorded in order to match the two groups. IQ was assessed using the two-subtest version (Vocabulary and Matrix Reasoning) of the Wechsler Abbreviated Scale of Intelligence—Second UK Edition (WASI-II^UK^; Wechsler 2011). There was no significant difference between groups for gender, age, ethnicity, IQ and socio-economic status (Table 1).

**Table 1:** Group differences on baseline measures and scores on measures of anxiety, depression and inattention

	CTD (n = 29)	Control (n = 25)	Group comparison (CTD, control)
Age	11.28 (1.68)	11.17 (2.46)	t(52) = 0.98, p > 0.05
Gender	14 females (48%)	12 females (48%)	χ^2^(1) = 0.75, p > 0.05
Ethnicity	25 White British (86%)	20 White British (80%)	χ^2^(5) = 0.66, p > 0.05
IQ	109.360 (12.93)	112.36 (10.75)	t(52) = − 1.05, p > 0.05
Anxiety	50.62 (10.14)	44.92 (7.99)	*t*(52) = 2.47, p < 0.05, *r* = 0.32
Depression	52.86 (8.31)	49.96 (8.06)	*t*(52) = 1.30, p > 0.05
Inattention	25.38 (6.89)	16.43 (4.14)	*t*(51) = 5.59, p < 0.001, *r* = 0.61

Means and standard deviations reported are prior to transformation to enable ease of interpretation

Participants were recruited via advertisements on Tourette’s Action volunteer webpage, King’s College London volunteer webpage, the Tics and Neurodevelopmental Movements Service (TANDeM) at Evelina London Children’s Hospital and schools in London. The Psychiatry, Nursing and Midwifery Research Ethics Committee at King’s College London and from the National Research Ethic Service Committee South Central-Oxford C gave ethical approval. The study was completed in accordance with the Declaration of Helsinki (7th revision, 2013). All participants and their parents provided informed consent.

Exclusion criteria included being outside of the age range 8–19; not being able to understand and complete the consent form and measures; and participants with any other diagnosed neurological condition or diagnosed learning disability. A diagnosis of CTD or TS was necessary for inclusion in the CTD group. For the control group, participants were excluded if they had a diagnosis of CTD or displayed significant tics. In an initial interview with the first author of the study (who is a trained clinician), parents were asked brief screening questions to list any medical (including psychiatric and neurological) diagnoses that the young person had received. Due to the well-established high rates of co-morbidity in CTD, participants with comorbid neurodevelopmental (providing that they were able to understand the consent process and measures) or psychiatric diagnoses were not excluded. A formal diagnostic assessment was not conducted due to time constraints.

### Tic-Specific Measures

#### Yale Global Tic Severity Scale (YGTSS)

The YGTSS (Leckman et al. 1989) assesses tic 1severity and is administered as a clinical interview with the young person and their parent. The YGTSS has good internal consistency and inter-rater reliability, as well as good convergent, divergent and discriminant validity (Leckman et al. 2006). In the current study an experienced clinician (Removed for Blind Review, clinical psychologist) rated the YGTSS, with a subsample (n = 14) co-rated by trained undergraduate students demonstrating good inter-rater reliability (94% agreement).

#### Premonitory Urge for Tics Scale (PUTS)

The PUTS (Woods et al. 2005) is a nine-item self-report questionnaire assessing premonitory experiences prior to tic onset. The PUTS demonstrates good internal consistency and test–retest reliability, as well as good construct and concurrent validity (Reese et al. 2014; Woods et al. 2005). In the present study, internal consistency was good (Cronbach’s alpha = 0.82).

#### Gilles de la Tourette Quality of Life scale (GTS-QoL)

The GTS-QoL (Cavanna et al. 2008) is a 27-item disease specific self-report questionnaire measuring quality of life. Good internal consistency, test–retest reliability and convergent validity have been demonstrated (Cavanna et al. 2008). Internal consistency was excellent (Cronbach’s alpha = 0.91) in this study.

### Measures of Clinical Co-morbidities

#### Revised Child Anxiety and Depression Scale (RCADS)

The RCADS (Chorpita et al. 2005) is a 47 item self-report questionnaire measuring anxiety and depression. Summed scores are converted into standardised T-scores for the young person’s age. The RCADS demonstrates high internal consistency and convergent validity (Chorpita et al. 2005; Ebesutani et al. 2011). In the current study, the internal consistency for the anxiety scale was excellent (Cronbach’s alpha = 0.92), but questionable for the depression scale (Cronbach’s alpha = 0.63).

#### Inattention Subscale of the Swanson, Nolan, and Pelham, Version IV Rating Scale (SNAP-IV)

The SNAP-IV (Swanson 1992) is a reliable and valid measure of ADHD symptoms that has three subscales: inattention, hyperactivity and opposition defiance (Bussing et al. 2008; Swanson et al. 2001). The internal consistency for the inattention subscale was excellent in this study (Cronbach’s alpha = 0.93).

### Experimental Measures

#### Heartbeat Counting Task

This is a standard Mental Tracking Paradigm designed to measure IA (Schandry 1981). Participants were asked to count their heartbeats (*counted heartbeats*) during two conditions (*HBP Baseline* and *HBP Manipulation*) of three separate trials (35, 25 and 45 s). *Condition 1 (HBP Baseline)* participants were given standard instructions that were consistent with previous research (“count all the heartbeats you feel in your body”; Dunn et al. 2007; Eley et al. 2004; Garfinkel et al. 2015). *Condition 2 (HBP Manipulation)* participants were instructed to inhibit their movements and count their heartbeats (“Now what I would like you to do, is to do your best not to move your body at all whilst you count your heartbeats”). In both conditions, participants were asked not to use any pulse-taking methods or employ strategies such as holding their breath. Movement frequency was monitored by the researchers as a manipulation check, which was measured using a count of the number of movements the young person made during the trials, including tics. To ensure that condition 1 was consistent with previous protocols, all trials for condition 1 preceded condition 2. There was a 20-s break in between each trial and a two-minute break between each condition. Apart from the instruction, there were no differences between condition 1 and condition 2.

The *actual number of heartbeats* was recorded with Polar RCS800CX. Polar Pro Trainer 5 software was used to analyse the data. Accuracy for each condition was calculated using:

$$1/3\sum {\left[ {1 - (\left| {{\text{actual heartbeats}} - {\text{counted heartbeats}}} \right|/{\text{actual heartbeats}})} \right]} .$$

### Procedure

Researchers met participants individually to complete the testing session. Tasks were administered in a set order, with the researcher present throughout. First participants completed the WASI-II^UK^, then the questionnaire measures, next the HBP task and finally the YGTSS as part of a larger experimental battery, which included two other experimental cognitive tasks (unrelated to the present study). Participants were reimbursed for their time (£15) and any travel expenses they incurred.

### Data Analysis

Anxiety, depression and inattention scores were compared across groups. The anxiety data were not normally distributed and so reciprocal transformations were applied. When there were significant group differences between these clinical variables, they were included as covariates in further analyses. To investigate group effects on HBP in the two conditions, a mixed-measures ANCOVA with Condition (HBP Baseline; HBP Manipulation) as the within-subject variable and group (CTD; control) as the between-subject variable was performed. As comorbidity and medication use in the CTD group has the potential to impact on group differences, we also replicated this analysis excluding participants (1) with comorbidity and (2) currently taking medication. Another mixed-measures ANOVA was performed to check the manipulation, with movement frequency per Condition as the within-subjects factor and Group as the between-subjects factor. HBP data was inspected for outliers and data points (n = 3) more than three standard deviations above the mean were removed. For data that did not meet distributional assumptions, bootstrapping^2^ was used for inference.

To examine the relationships between HBP and PU in the CTD group, partial correlations were computed, while controlling for tic severity. Regression analysis was then used to investigate the relationship between HBP and anxiety. To investigate whether this relationship varied by group, moderation analysis using multiple regression was performed. Continuous variables were mean-centred and the product of the two predictor variables (group × HBP) were entered as an interaction term. Correlation analyses were used to follow-up any significant findings. Partial correlations, controlling for tic severity, were then used to investigate the association between HBP and quality of life in the CTD group.

## Results

Independent t-tests revealed a main effect of Group (CTD, control) on anxiety and inattention scores but not depression scores, with the CTD group reporting more symptoms of each (Table 1). As anxiety and inattention differed across groups, these variables were included as covariates in subsequent analyses. Inattention data was missing for one control participant, excluding them from relevant analyses. Inattention did not correlate significantly with anxiety [*r*(51) = 0.26, p = 0.058] or accuracy scores in Condition 1 [*r*(51) = − 0.068, p = 0.63] but inattention did significantly correlate with accuracy in Condition 2 [*r*(51) = − 0.35, p = 0.01].

### HBP in Youth with CTD versus Typically Developing Controls

The mixed measures ANCOVA for HBP revealed a main effect of Condition and significant Group-by-Condition and Inattention-by-Condition interactions (Table 2). The Group-by-Condition interaction was followed-up using ANOVAs for each condition separately. [Inattention and anxiety were included as covariates in this analysis, anxiety was then removed as it had no significant effect on accuracy across conditions.] This revealed a significant difference in accuracy between the groups for condition 1 (HBP Baseline) only [*F*(1, 52) = 4.55, p = 0.038, $\eta _{p}^{2}$= 0.083], where the CTD group ($\bar{\rm x}$ = 0.58; SD = 0.22) were less accurate than controls ($\bar{\rm x}$ = 0.72; SD = 0.22). There was no significant difference between the groups for Condition 2 [F(1,52) = 0.22, p = 0.64; CTD group: $\bar{\rm x}$ = 0.63; SD = 0.20; control group: $\bar{\rm x}$ = 0.66; SD = 0.21). [Of note, the same pattern of results is found when inattention is not included as a covariate.] The Inattention-by-Condition interaction was followed-up by calculating difference scores in accuracy across the conditions. This revealed a significant relationship between inattention and change in accuracy over the two conditions (r(51) = − 0.30, p = 0.032), with those reported as being more inattentive showing smaller increases in accuracy from condition 1 to 2.

**Table 2:** Changes in heartbeat perception accuracy and movement frequency following an instruction to inhibit movement (Condition 2: HBP manipulation)

	F	P	$\eta _{p}^{2}$
Accuracy			
Condition (condition 1/condition 2)	*F*(1,50) = 8.57	0.005	0.15
Condition × inattention	*F*(1,50) = 9.28	0.004	0.16
Condition × group	*F*(1,50) = 4.16	0.047	0.077
Between subjects effect of group	*F*(1,49) = 2.12	0.15	
Movement			
Condition (condition 1/condition 2)	F(1,50) = 0.011	0.92	
Condition × inattention	F(1,50) = 0.94	0.34	
Condition × group	F(1,50) = 6.49	0.014	$\eta _{p}^{2}$ = 0.12
Between subjects effect of group	F(1,50) = 4.52	0.038	$\eta _{p}^{2}$ = 0.083

To check the impact of comorbid diagnoses on group differences, this analysis was rerun excluding participants with diagnoses of ADHD, OCD and/or ASD, resulting in 21 participants in the CTD group. The same pattern of results was observed with main effects of Condition, F(1,42) = 7.77, p = 0.008, $\eta _{p}^{2}$ = 0.16, and significant Group-by-Condition, F(1,42) = 7.09, p = 0.011, $\eta _{p}^{2}$ = 0.14 and Inattention-by-Condition interactions, F(1,42) = 5.56, p = 0.023, $\eta _{p}^{2}$ = 0.12. The Group-by-Condition interaction was driven by a significant difference in accuracy between the groups for condition 1 (*F*(1, 42) = 5.99, p = 0.019, $\eta _{p}^{2}$ = 0.12), with no significant difference between the groups for Condition 2 (F(1,42) = 2.45, p = 0.13). The same pattern of results was also observed when young people who were currently taking medication were excluded, leaving a sample of n = 23 in CTD group [main effects of Condition, F(1,44) = 4.85, p = 0.033, $\eta _{p}^{2}$= 0.099; significant Group-by-Condition interaction, F(1,44) = 4.45, p = 0.042, $\eta _{p}^{2}$= 0.092; significant Inattention-by-Condition interaction, F(1,44) = 4.15, p = 0.086, $\eta _{p}^{2}$= 0.086; significant difference in accuracy between the groups for condition 1 (*F*(1,44) = 4.71, p = 0.035, $\eta _{p}^{2}$= 0.097) but not Condition 2 (F(1,44) = 0.28, p = 0.60)].

The repeated-measures ANOVA with movement frequency revealed a main effect of Group and a significant Group-by-Condition interaction (Table 2). Follow-up analyses using difference scores in movements revealed larger *increases* in movements in the CTD group compared to controls, t(52) = 4.34, p < 0.01 (CTD: $\bar{\rm x}$ = 3.17, SD=4.00; control: $\bar{\rm x}$ = 0.52, SD = 1.50). A single item measure of tic suppression (PUTS item 10) was significantly correlated with increased movements, r(27) = − 0.39, p < 0.05, wherein those who rated themselves as worse at suppressing tics displayed more movements from condition 1 (HBP Baseline) to condition 2 (HBP Manipulation). Movement frequency in the control group was very low across conditions (condition 1: $\bar{\rm x}$ = 1.16; SD = 1.82; condition 2: $\bar{\rm x}$ = 0.76; SD = 1.85). Movement frequency in the CTD group was also fairly low in condition 1 ($\bar{\rm x}$ = 0.1.93; SD = 2.43) and rose considerably in condition 2 ($\bar{\rm x}$ = 0.5.10; SD = 4.56).

### Clinical Features and HBP in Youth with CTD

HBP in condition 1 (HBP Baseline) did not significantly correlate with PU when tic severity was controlled for [r(26) = 0.11, p = 0.56]. Regression analysis with anxiety as the dependent variable and Group, HBP Baseline, depression and inattention as predictor variables was significant [*F*(4,52) = 12.20, p < 0.001, R^2^ = 0.50], but indicated that there was no main effect of HBP on anxiety across groups and only depression significantly predicted anxiety score (Table 3). Moderation analysis, including depression as a covariate, revealed a significant interaction between Group and HBP (*F*(4,53) = 13.65, *p* < 0.001, R^2^ = 0.53 ∆R^2^ = 0.038; see Table 3 for full analysis; inattention was controlled for initially and then removed as it was non-significant in previous analysis). Follow-up analysis revealed HBP significantly correlated with anxiety in the CTD group, *r*(27) = 0.40, p = 0.032 but not the control group, *r*(23) = − 0.13, p = 0.54. Partial correlations (controlling for tic severity) were then used to investigate the relationship between quality of life and HBP in condition 1, this revealed a significant positive relationship [r(26) = 0.39, p = 0.041].

**Table 3:** Regression analysis to investigate whether the relationship between heartbeat perception (HBP) and anxiety and whether this is moderated by group

Predictors	B	SE B	β	*p*	95% CI
Model 1					
Constant	0.036	0.004			
Group	0.001	0.001	0.13	0.30	− 0.01 to 0.003
HBP condition 1	0.001	0.002	0.041	0.70	− 0.003 to 0.005
Depression	0.00	0.00	− 0.66	0.001	0.00–0.00
Inattention	0.00	0.00	− 0.068	0.63	0.00–0.00
Model 2					
Constant	0.034	0.003			
Group	0.002	0.001	− 0.20	0.087	0.00–0.003
HBP condition 1	0.001	0.002	− 0.065	0.54	− 0.003 to 0.006
Depression	0.00	0.00	0.64	0.001	0.00–0.00
Step 2					
Constant	0.042	0.005			
Group	0.001	0.001	− 0.17	0.11	0.00–0.003
HBP condition 1	− 0.01	0.006	0.514	0.068	− 0.022 to 0.001
Depression	0.00	0.00	0.64	0.00	0.00–0.00
HBP × group	0.002	0.001	− 0.63	0.045	0.0001–0.004

Bootstrapped standard errors, p-vales and 95% confidence intervals are reported

*SE* standard error; *CI* confidence interval; *HBP* heartbeat perception

## Discussion

Theoretical interest in altered IA in both CTD (Ganos et al. 2015) and anxiety disorders (e.g. Domschke et al. 2010) has been growing independently. This is the first study to connect the two fields and investigate HBP as a common factor for anxiety and PU in youth with CTD. This study has three main sets of findings relating to, (1) HBP when measured traditionally, (2) experimental manipulation of HBP using an instruction to inhibit movements and (3) the relationship between HBP and clinical features of youth with CTD. Consistent with predictions, youth with CTD had reduced IA compared to controls when measured traditionally (even when participants with co-morbidity/medication use were excluded). In response to the experimental manipulation, IA increased so that the groups were no longer significantly different. Finally, in the CTD group, increased HBP was associated with more anxiety symptoms and reduced quality of life (but not PU, depression or inattention).

Our finding that youth with CTD had lower HBP compared to controls (even when comorbidities were controlled for) replicates and extends previous findings in adults with CTD (Ganos et al. 2015). Reduced IA may therefore reflect an underlying trait in CTD, although it is unclear whether this trait precedes tic onset or arises as a compensatory mechanism to help the person manage the tics. In contrast to the adult study, we did not replicate the finding that HBP was associated with PU, which suggests that the internal perception of sensory phenomena differs across the age span for people with CTD. This may reflect age-associated changes in PU, as research indicates that awareness of PU increases with age (Banaschewski and Rothenberger 2003). However, whether this age related change reflects a stronger PU, increased IA or a combination of these and other factors is unclear. Performance on a more PU-specific measure of IA, such as muscle tension, and multiple measures of PU (e.g. clinician administered; McGuire et al. 2016) would further understanding of whether there is a reliable age-related relationship between these variables.

Previous attempts to experimentally alter HBP in the general population have been mixed (Ainley et al. 2012, 2013; Fairclough and Goodwin 2007; Stevens et al. 2011) and this is the first study to demonstrate that it is possible to experimentally manipulate HBP in youth with CTD. Psychological therapies for tic disorders assume that it is possible to modify an individual’s internal awareness (Van de Griendt et al. 2013). Thus, our finding that HBP was lower in the CTD group and that accuracy increased with the instruction to inhibit movement, supports this premise, as well as the notion that IA can be influenced by state factors (Ainley et al. 2012). Interestingly, but perhaps unsurprisingly, youth with higher levels of inattention exhibited smaller increases in IA and inattention correlated with IA in the second condition. This is consistent with research identifying that comorbid ADHD reduces treatment response to behavioural therapy for tics (McGuire et al. 2014) and reinforces the need to consider an individual’s level of cognitive control and attentional processing style when delivering tic treatments. The increase in movements for youth with CTD in response to the instruction to ‘inhibit’ movements is also of interest as it highlights how direct instruction to inhibit can actually exacerbate movements. It is speculated that this may have arisen due to increased allocation of attention to movement and accompanying tic-related cognitions, which previous research has shown can increase tic frequency (Brandt et al. 2014; Misirlisoy et al. 2015; O’Connor et al. 2014). Exploration of cognitive aspects of interoception (i.e. interoceptive sensibility and awareness) would be of interest to help elucidate the relationship between movements, internal bodily sensations and tic-related cognitions for individuals with CTD.

In relation to clinical features, increased HBP was associated with increased anxiety symptoms and reduced quality of life for youth with CTD.^3^ Aberrant HBP is implicated in the development and maintenance of anxiety during typical development and this current finding is consistent with that research (Eley et al. 2004, 2007). The association between HBP and quality of life suggests that for youth with CTD increased awareness of internal bodily sensations is related to a general reduction in well-being and satisfaction with life. Given that enhanced HBP is associated with anxiety and that youth with CTD are at an increased risk of developing anxiety and lower quality of life, the finding that those with CTD exhibited lower levels of IA than controls is perhaps unexpected (albeit consistent with adults with CTD). These results require replication but highlight the importance of considering co-morbidities in research on tics and in the treatment of youth with CTD, with IA offering a potential transdiagnostic treatment target.

Habit Reversal Therapy (HRT), a leading evidence-based psychological therapy for tic disorders, emphasises the need to increase awareness of internal sensations to learn when tics are likely to occur (Van de Griendt et al. 2013). The current findings suggest a secondary outcome for tic-related therapies could be to learn that these internal sensations are not threatening (i.e. they no longer signal tic onset), similar to cognitive models for anxiety disorders. Perhaps an important difference between HRT and cognitive therapy for anxiety disorders is that whilst HRT aims (in part) to enhance internal awareness, cognitive therapy for anxiety can include training to shift attentional focus from internal sensations to the external environment. Cognitive behavioural and metacognitive techniques that encourage attentional control and flexible attentional shifting could be a useful addition to current therapies for tic disorders (Robinson and Hedderly 2016). Importantly, what our data cannot determine is whether an underlying interoceptive ability has the same relationship with anxiety as taught awareness (such as in psychological therapies); it would be informative for future research to track changes in IA during treatment.

This current study had limitations. Firstly, the data are cross-sectional and so causal effects cannot be determined, with a need for longitudinal studies to understand the direction of the links between HBP and anxiety, as well as whether HBP does change with therapy. Secondly, we did not replicate findings linking HBP and PU. This is likely to be due to age differences, as such studies that use multiple age groups and larger samples would be helpful. Thirdly, the sample did not have diagnoses of anxiety disorders, so, to disentangle the relationships between HBP, anxiety and CTD, it may be valuable to compare a sample of youth with CTD and an anxiety disorder to with those with CTD without anxiety disorders, and those with anxiety disorders without CTD. Adding to this, some research suggests that OCD presents differently in youth with Tourette syndrome, compared to those with chronic motor or vocal tic only (Diniz et al. 2006), so it may also be important to distinguish these groups when investigating relationships with anxiety.

There are also several limitations in terms of our sample, including the small sample size, diagnostic comorbidity and use of medication. Given that both comorbidity and medication use is high in the CTD population (Kumar et al. 2016), it did not seem representative to exclude participants with comorbidity or medication use. Although both could impact on task performance (for example difficulties with attention), group differences in HBP remained when participants with comorbidity/medication use were excluded and inattention was controlled for in principal analyses. Future studies with larger sample sizes (enabling detailed subgroup analysis) would permit a more thorough investigation of the impact that comorbidities and medication could have on interceptive accuracy. It would also be valuable for future studies to investigate the role of attention processes on IA in children with CTD. Furthermore, it is possible that there are differences between those participants who overestimate and underestimate their heart rate, and that the relationships vary within these groups with anxiety and PU. Larger samples that could evaluate relationships within subgroups would be helpful to establish whether these findings are consistent across performance or specific to those that underestimate or overestimate their heart rate. Finally, although our analysis was hypothesis driven, we did conduct multiple analyses and did not include statistical corrections for multiple testing. However, a two-tailed level of significance was used throughout, due to the exploratory nature of this study.

In summary, despite the limitations of the study, these data demonstrate a relationship between enhanced HBP and anxiety and lower quality of life in youth with CTD. They indicate that, like adults, HBP is reduced in youth with CTD compared to controls, with the novel finding that HBP can be experimentally enhanced in this group. These results highlight important questions for treatment development for youth with CTD and comorbid anxiety, with interoception offering a novel treatment target.

## Footnotes

1 Tic-specific measures were administered to participants with CTD only.

2 Bootstrapping is a computer-intensive, non-parametric approach to statistical inference that provides valid standard errors, confidence intervals and p values for hypothesis tests. It only assumes that the sampled data provide a reasonable representation of the population from which they came and therefore do not have to meet distributional assumptions (Davison & Hinkley, 2006).

3 Quality of life was only assessed in the CTD group as we used a tic specific measure.

## References

[CR1] Ainley V, Maister L, Brokfeld J, Farmer H, Tsakiris M (2013). More of myself: Manipulating interoceptive awareness by heightened attention to bodily and narrative aspects of the self. Consciousness and Cognition.

[CR2] Ainley V, Tajadura-Jimenez A, Fotopoulou A, Tsakiris M (2012). Looking into myself: Changes in interoceptive sensitivity during mirror self-observation. Psychophysiology.

[CR3] Banaschewski T, Rothenberger A (2003). Premonitory sensory phenomena and suppressibility of tics in Tourette syndrome: Developmental aspects in children and adolescents. Development and Psychopathology.

[CR4] Bitsko RH, Holbrook JR, Visser SN, Mink JW, Zinner SH, Ghandour RM, Stephen J (2015). A national profile of Tourette syndrome, 2011–2012. Journal of Developmental and Behavioral Pediatrics.

[CR5] Bloch MH, Leckman JF (2009). Clinical course of Tourette syndrome. Journal of Psychosomatic Research.

[CR6] Brandt VC, Lynn MT, Obst M, Brass M, Münchau A (2014). Visual feedback of own tics increases tic frequency in patients with Tourette’s syndrome. Cognitive Neuroscience.

[CR7] Bussing R, Fernandez M, Harwood M, Hou W, Garvan CW, Swanson JM, Eyberg SM (2008). Parent and teacher SNAP-IV ratings of attention deficit/hyperactivity disorder symptoms: Psychometric properties and normative rating from a school district sample. Assessment.

[CR8] Cavanna AE, Schrag A, Morley D, Orth M, Robertson MM, Joyce E (2008). The Gilles de la Tourette Syndrome-Quality of Life Scale (GTS-QOL): Development and validation. Neurology.

[CR9] Chorpita BF, Moffitt CE, Gray J (2005). Psychometric properties of the Revised Child Anxiety and Depression Scale in a clinical sample. Behaviour Research and Therapy.

[CR10] Conelea C, Woods DW, Zinner SH, Budman C, Murphy T, Scahill LD (2011). Exploring the impact of chronic tic disorders on youth: Results from the Tourette syndrome impact survey. Child Psychiatry and Human Development.

[CR11] Craig AD (2003). Interoception: The sense of the physiological condition of the body. Current Opinion in Neurobiology.

[CR12] Crossley E, Cavanna AE (2013). Sensory phenomena: Clinical correlates and impact on quality of life in adult patients with Tourette syndrome. Psychiatry Research.

[CR13] Diniz JB, Rosario-Campos MC, Hounie AG, Curi M, Shavitt RG, Lopes AC, Miguel EC (2006). Chronic tics and Tourette syndrome in patients with obsessive-compulsive disorder. Journal of Psychiatric Research.

[CR14] Domschke K, Stevens S, Pfleiderer B, Gerlach AL (2010). Interoceptive sensitivity in anxiety and anxiety disorders: An overview and integration of neurobiological findings. Clinical Psychology Review.

[CR15] Dunn BD, Dalgleish T, Ogilvie AD, Lawrence AD (2007). Heartbeat perception in depression. Behaviour Research and Therapy.

[CR16] Dunn BD, Stefanovitch I, Evans D, Oliver C, Hawkins A, Dalgleish T (2010). Can you feel the beat? Interoceptive awareness is an interactive function of anxiety and depression specific symptom dimensions. Behaviour Research and Therapy.

[CR17] Eapen V, Snedden C, Črnčec R, Pick A, Sachdev P (2016). Tourette syndrome, co-morbidities and quality of life. Australian & New Zealand Journal of Psychiatry.

[CR18] Ebesutani C, Chorpita BF, Higa-Mcmillan CK, Nakamura BJ, Regan J, Lynch RE (2011). A psychometric analysis of the revised child anxiety and depression scales-parent version in a school sample. Journal of Abnormal Child Psychology.

[CR19] Eley TC, Gregory AM, Clark DM, Ehlers A (2007). Feeling anxious: A twin study of panic/somatic ratings, anxiety sensitivity and heartbeat perception in children. Journal of Child Psychology and Psychiatry and Allied Disciplines.

[CR20] Eley TC, Stirling L, Ehlers A, Gregory AM, Clark DM (2004). Heart-beat perception, panic/somatic symptoms and anxiety sensitivity in children. Behaviour Research and Therapy.

[CR21] Fairclough SH, Goodwin L (2007). The effect of psychological stress and relaxation on interoceptive accuracy: Implications for symptom perception. Journal of Psychosomatic Research.

[CR22] Ganos C, Garrido A, Navalpotro-Gómez I, Ricciardi L, Martino D, Edwards MJ (2015). Premonitory urge to tic in Tourette’s is associated with interoceptive awareness. Movement Disorders.

[CR23] Garfinkel SN, Seth AK, Barrett AB, Suzuki K, Critchley HD (2015). Knowing your own heart: Distinguishing interoceptive accuracy from interoceptive awareness. Biological Psychology.

[CR24] Hesapçıoğlu ST, Tural MK, Kandil S (2014). Quality of life and self-esteem in children with chronic tic disorder. Turk Pediatri Arsivi.

[CR25] Jackson SR, Parkinson A, Kim SY, Schüermann M, Eickhoff SB (2011). On the functional anatomy of the urge-for-action. Cognitive Neuroscience.

[CR26] Khalsa SS, Lapidus RC (2016). Can interoception improve the pragmatic search for biomarkers in psychiatry?. Frontiers in Psychiatry.

[CR27] Khalsa SS, Rudrauf D, Damasio AR, Davidson RJ, Lutz A, Tranel D (2008). Interoceptive awareness in experienced meditators. Psychophysiology.

[CR28] Kumar A, Trescher W, Byler D (2016). Tourette syndrome and comorbid neuropsychiatric conditions. Current Developmental Disorders Reports.

[CR29] Kwak C, Dat Vuong K, Jankovic J (2003). Premonitory sensory phenomenon in Tourette’s syndrome. Movement Disorders.

[CR30] Leckman JF, Bloch MH, Scahill L, King RA (2006). Tourette syndrome: The self under siege. Journal of Child Neurology.

[CR31] Leckman JF, Riddle MA, Hardin MT, Ort SI, Swartz KL, Stevenson JO, Cohen DJ (1989). The Yale Global Tic Severity Scale: Initial testing of a clinician-rated scale of tic severity. Journal of the American Academy of Child & Adolescent Psychiatry.

[CR32] Lewin AB, Storch EA, Conelea CA, Woods DW, Zinner SH, Budman CL (2011). The roles of anxiety and depression in connecting tic severity and functional impairment. Journal of Anxiety Disorders.

[CR33] McGuire JF, McBride N, Piacentini J, Johnco C, Lewin AB, Murphy TK, Storch EA (2016). The premonitory urge revisited: An individualized premonitory urge for tics scale. Journal of Psychiatric Research.

[CR34] McGuire JF, Piacentini J, Brennan EA, Lewin AB, Murphy TK, Small BJ, Storch EA (2014). A meta-analysis of behavior therapy for Tourette syndrome. Journal of Psychiatric Research.

[CR35] Misirlisoy E, Brandt V, Ganos C, Tübing J, Münchau A, Haggard P (2015). The relation between attention and tic generation in Tourette syndrome. Neuropsychology.

[CR36] O’Connor K, St-Pierre-Delorme M-E, Leclerc J, Lavoie M, Blais MT (2014). Meta-cognitions in tourette syndrome, tic disorders, and body-focused repetitive disorder. Canadian Journal of Psychiatry. Revue canadienne de psychiatrie.

[CR37] Pollatos O, Traut-Mattausch E, Schandry R (2009). Differential effects of anxiety and depression on interoceptive accuracy. Depression and Anxiety.

[CR38] Reese HE, Scahill L, Peterson AL, Crowe K, Woods DW, Piacentini J (2014). The premonitory urge to tic: Measurement, characteristics, and correlates in older adolescents and adults. Behavior Therapy.

[CR39] Robinson S, Hedderly T (2016). Novel psychological formulation and treatment of “Tic Attacks” in Tourette syndrome. Frontiers in Pediatrics.

[CR40] Schandry R (1981). Heart beat perception and emotional experience. Psychophysiology.

[CR41] Stevens S, Gerlach AL, Cludius B, Silkens A, Craske MG, Hermann C (2011). Heartbeat perception in social anxiety before and during speech anticipation. Behaviour Research and Therapy.

[CR42] Swanson JM (1992). School-based assessments and interventions for ADD students.

[CR43] Swanson JM, Kraemer HC, Hinshaw SP, Arnold LE, Conners CK, Abikoff HB (2001). Clinical relevance of the primary findings of the MTA: Success rates based on severity of ADHD and ODD symptoms at the end of treatment. Journal of the American Academy of Child and Adolescent Psychiatry.

[CR44] Van de Griendt JMTM, Verdellen CWJ, van Dijk MK, Verbraak MJPM (2013). Behavioural treatment of tics: Habit reversal and exposure with response prevention. Neuroscience and Biobehavioral Reviews.

[CR45] Wechsler D (2011). Wechsler Abbreviated Scale of Intelligence (WASI-II) (2nd ed.).

[CR46] Woods DW, Piacentini J, Himle MB, Chang S (2005). Premonitory urge for Tics Scale (PUTS): initial psychometric results and examination of the premonitory urge phenomenon in youths with tic disorders. Journal of Developmental and Behavioral Pediatrics: JDBP.

